# Effect of Ultrasonic Rolling on the Surface Integrity and High Temperature Oxidation Properties of Laser Melted NiCrAl Coatings

**DOI:** 10.3390/ma18174119

**Published:** 2025-09-02

**Authors:** Dejiang Zhang, Chengchao Hu, Xubo Liu, Dewei Hu, Ting Yu, Jiaming Zhan

**Affiliations:** 1 Advanced Manufacturing College, Nanchang University, Nanchang 330031, China; 19850675155@163.com (D.Z.); 18357309802@163.com (C.H.); 15607963216@163.com (D.H.);; 2 School of Advanced Manufacturing, Sun Yat-sen University, Shenzhen 518107, China

**Keywords:** NiCrAl coatings, USRP, surface integrity, residual stress, high temperature oxidation property

## Abstract

This study investigates the effects of ultrasonic surface rolling process (USRP) parameters—static load, indentation depth—on the surface integrity, mechanical properties, and high-temperature oxidation properties of laser-clad NiCrAl coatings. Comprehensive experimental results demonstrate that USRP treatment effectively eliminates surface cracks and significantly enhances surface integrity. The process simultaneously improves mechanical properties, with microhardness increasing by 24.6% due to grain refinement-induced strengthening and elevated dislocation density. Under constant preload, increasing the ultrasonic rolling indentation depth effectively transforms residual tensile stress into beneficial compressive stress (from +69.8 MPa to −1315.3 MPa), with higher preload further enhancing this effect. Moreover, USRP-treated coatings achieve complete oxidation resistance at elevated temperatures by forming a denser and more continuous oxide layer while effectively suppressing internal oxidation, resulting in markedly improved high-temperature oxidation performance. Quantitative analysis confirms that the enhancement in surface mechanical properties is primarily attributed to microstructural refinement and dislocation strengthening mechanisms.

## 1. Introduction

With the rapid advancement of aviation technology, the service temperature of the current engine is increasing, and some have even reached more than 1100 °C [1]. Under such harsh conditions involving high temperatures, pressures, and high-speed working environments [2], the conventional titanium alloys and aluminum-based alloys often fail to meet the use requirements [3,4,5,6]. To solve this problem, applying high-temperature-resistant coatings on the original high-temperature alloys is a relatively efficient method [7]. In aerospace engineering, anti-high-temperature oxidation coatings are widely used in the key components of engines [8]. Their main purpose is to enhance the oxidation resistance of high-temperature alloy parts [9]. Therefore, this type of coating needs to possess the ability to withstand high temperatures and resist high-temperature oxidation [10]. Moreover, it should have the basic characteristics of stable chemical properties and being resistant to decomposition under high-temperature conditions.

Among various coating materials, coated NiCrAl alloys [11,12,13,14] have been extensively investigated due to their excellent oxidation resistance and mechanical properties. However, such coatings are often found to fail in practical applications due to the obvious difference between the microstructure and the substrate [15], whereas the laser cladding technology offers a promising alternative by enabling metallurgical bonding and in situ reinforcement [16,17]. Chen et al. [18] conducted the microstructure study on (TiO_2_ + B_2_O_3_ + Al_2_O_3_ + TiB_2_)/NiCrAl cermet coatings prepared on the surface of 40Cr steel using laser cladding technology. Experimental studies showed that TiB_2_ and Al_2_O_3_ ceramic particles contributed to internal grain reinforcement, but the coating exhibits poor thermal conductivity and is prone to deformation during processing, making it difficult to guarantee the quality of the alloy coating after processing. To avoid this situation, various mechanical surface treatments have been explored, such as shot peening [19], deep cold rolling [20], grinding [21], ultrasonic rolling strengthening [22], ultrasonic polishing [23], and other technical means without changing the chemical composition of the material, among which rolling technology is the most widely used. The corrosion resistance, oxidation resistance, abrasion resistance, and erosion resistance of metal materials largely depend on their surface integrity [24]. Ultrasonic surface rolling process is a kind of processing method that uses the high-frequency vibration of ultrasonic and exerts a certain pressure on the surface of the workpiece through the rolling tool at ambient temperature to produce a small plastic deformation, so as to improve its surface quality and performance. Yao et al. [25] studied the change law of the surface integrity of 718 nickel alloy during the process of turning and ultrasonic rolling strengthening, and found that after ultrasonic rolling, the dislocation density increased rapidly, and the grain size was refined, which greatly improved the wear resistance and fatigue strength of the alloy surface. Similarly, Yu et al. [26] demonstrated that USRP treatment of GH4169 alloy reduced surface roughness from 1.17 μm to 0.08 μm, and the microhardness and fatigue resistance of the material were significantly improved. Dang et al. [27] reported a 20–30% hardness increase via dislocation accumulation. Dang et al. [28] demonstrated that USRP-processed thermal spray coatings exhibit 50% longer oxidation life at 800 °C due to Al_2_O_3_ layer formation.

Despite these advances, the synergistic effects of USRP on laser-clad NiCrAl coatings—particularly concerning high-temperature oxidation resistance—remain underexplored. This study aims to bridge this knowledge gap by applying USRP to laser-clad NiCrAl coatings deposited on 45# steel. With technologies of SEM, EDS, metallographic electron microscope, 3D profile roughness meter, residual stress analyzer, surface hardness meter, and other instruments, the surface microstructure, cross-section morphology, and mechanical properties were analyzed, and the mechanism of surface strengthening and high-temperature oxidation resistance was discussed.

## 2. Experimental Details

### 2.1. Preparation of Materials

The substrate used in this study was 45# steel (international standard designation: C45E4), coated with NiCrAl alloy, and the chemical composition of the coating NiCrAl powder mixture is shown in Table 1. The circular coating specimens (70 mm diameter × 10 mm thickness) were fabricated by laser cladding technology with process parameters details in Table 2. The NiCrAl single-layer coatings prepared by 1200 W laser cladding power were selected as ultrasonic rolling test specimens in this paper.

### 2.2. Experimental Procedures

NiCrAl alloy powder was used as the raw material, with a chemical composition of 5 % Al, 17 % Cr, and bal Ni (wt%). Experiments in laser cladding were performed on a 45# steel substrate with dimensions of 70 mm × 70 mm × 10 mm, utilizing the 3D printing and laser remanufacturing platform complete set equipment (RC-LCD-4000-F-R laser system, Nanjing, China). Nitrogen was employed as the shielding gas at a flow rate of 15 L per minute. The laser processing parameters were as follows: laser power of 1 kW, circular spot with a diameter of 3.5 mm, scanning speed of 300 mm/min, and an overlap ratio of 50 %. After cladding, the nine specimens were set into three groups with sample 0 serving as the untreated control (no ultrasonic rolling). The specimens 1–4 were processed at a constant static load of 0.3 MPa with varying rolling depth (0.05 mm, 0.085 mm, 0.125 mm, and 0.15 mm, respectively). Similarly, specimens 5–8 were treated at an elevated static load of 0.4 MPa with varying depth (0.05 mm, 0.085 mm, 0.125 mm, and 0.15 mm).

The vibration frequency of the ultrasonic surface rolling equipment was set up to 30 kHz, the amplitude was 8 μm, the diameter of the scrollable ultrasonic surface rolling ball was 8 mm, the operating current was 1.0 A, the lateral feed (or step-over distance) between adjacent rolling paths was set to 2 mm, the surface coverage, which is defined as the percentage of the surface area that has undergone plastic deformation, was approximately 100%, and the static load range was 0.3–0.4 MPa (covering the threshold for severe plastic deformation, as defined >10% strain under 0.5 MPa in preliminary tests). During the USR process, the middle of the specimen is USR processed. Emulsion was used to cool the tool and the processing area during USR processes. Each specimen was USR processed once.

After USRP treatment, the microstructural characterization and elemental composition of the specimens were performed using a scanning electron microscope (Zeiss EVO18, Oberkochen, Germany; equipped with a tungsten filament and an energy-dispersive X-ray spectroscopy (EDS) detector). The surface roughness parameters were measured using three-dimensional profilometry (ContourG 3D Non-contact Optical profilometer, Bruker Corp., Tucson, AZ, USA). For measurement, the area of interest was first identified under a 2.5× objective lens, where the light intensity was adjusted for optimal clarity. Once the area was properly focused and centered, high-resolution surface topography data were acquired using a 10× objective lens. Microhardness measurements were conducted on the sample surfaces using a Vickers hardness tester (MVD-1000D1 Automatic turret digital display microhardness tester, Shanghai Jimin Measuring Equipment Co., Ltd., Shanghai, China) under a 200 gf load for 15 s. During mechanical testing, at least three samples were measured for each condition.

The surface residual stress along rolling direction on the top of the samples was measured using an X-ray diffractometer (XRD; Stress3000, Stresstech Oy, Vantaa, Finland) with Cr Kα radiation. For the stress test, the (311) diffraction plane was selected, with a 2θ scan range of −90–90°. Measurements were taken from at least three different areas per sample. According to the standard engineering sign convention, positive values (+) indicate tensile stress and negative values (−) indicate compressive stress.

For microstructural examination, samples were mechanically polished to mirror finish and electrolytically etched in a perchloric acid-ethanol solution (50 mL hydrochloric acid + 5 g copper sulfate + 50 mL water) for 30 s. Metallographic microstructure was measured using Metallographic embedding machine (XQ-1, Shanghai Shangcai Testing Machine Co., Ltd., Shanghai, China).

The high temperature oxidation resistance was measured using resistance furnace control box (SA2-4-14TP, Nanyang Xinyu New Material Technology Co., Ltd., Nanyang, China). The parameters were as follows: rated power of 2.5 kW, temperature fluctuation of 5 °C.

## 3. Coating Organizational Properties Testing

To prevent potential coating damage, the surface morphology of the specimens was characterized by a non-contact optical profilometer. The microstructural characterization and elemental composition analysis of the specimens were performed using scanning electron microscopy (SEM) equipped with energy-dispersive X-ray spectroscopy (EDS) detectors. The microhardness measurements were conducted using a Vickers microhardness tester (MVD-1000D1 Automatic turret digital display microhardness tester, Shanghai Jimin Measuring Equipment Co., Ltd., Shanghai, China). The isothermal oxidation test of the coating was performed at 800 °C for a total duration of 100 h, with intermediate measurements taken at four equally spaced time intervals.

### 3.1. Microstructure Analysis

**Figure 1:**
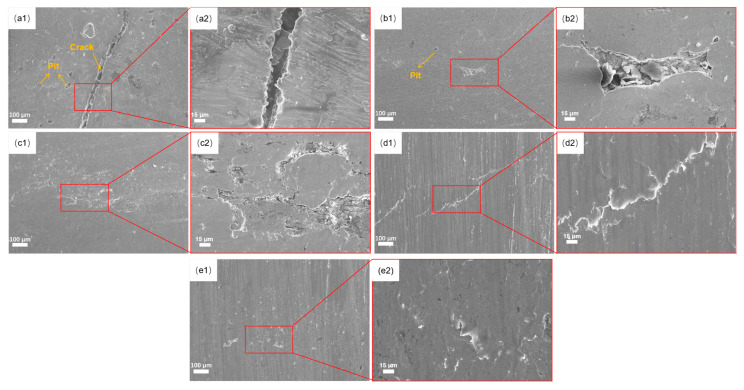
Surface morphology of coatings with different indentations at 0.3 MPa: (**a1**,**a2**) no ultrasonic rolling, (**b1**,**b2**) rolling depth of 0.05 mm, (**c1**,**c2**) rolling depth of 0.085 mm, (**d1**,**d2**) rolling depth of 0.125 mm, (**e1**,**e2**) rolling depth of 0.15 mm.

**Figure 2:**
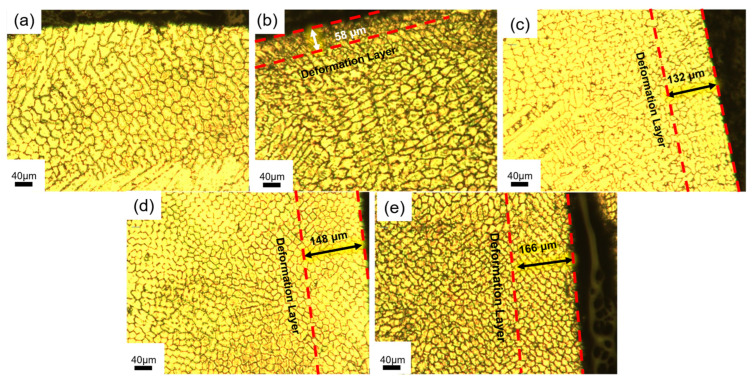
Metallographic microstructure of NiCrAl coatings at 0.3 MPa with different rolling depths: (**a**) no ultrasonic rolling, (**b**) rolling depth of 0.05 mm, (**c**) rolling depth of 0.085 mm, (**d**) rolling depth of 0.125 mm, (**e**) rolling depth of 0.15 mm.

Figure 1 presents the SEM micrograph (500×) of the specimen surface. The untreated coating (Figure 1a) exhibits numerous cracks, pits, and significant surface irregularities. These defects likely originate from insufficient melting of the powder material during laser cladding, which may be attributed to either an inadequate laser powder density or an excessive scanning speed. After USRP at a static load of 0.3 MPa, a notable grain refinement was observed in the coating (Figure 2b–e). Furthermore, as the rolling depth increased, the surface roughness decreased significantly, and crack formation was effectively suppressed.

Figure 2 illustrates the grain size distribution across the coating surface. The deformation layer was indicated by two red lines in Figure 2. The metallographic microstructure demonstrates that ultrasonic rolling effectively refines grains and eliminates surface cracks through severe plastic deformation. The smaller grains increase grain boundary area, dispersing plastic deformation across more grains. This reduces internal stress concentration and enhances intergranular bonding. As a result, crack propagation is effectively suppressed, leading to improved material toughness and strength.

### 3.2. Surface Elemental Analysis

**Figure 3:**
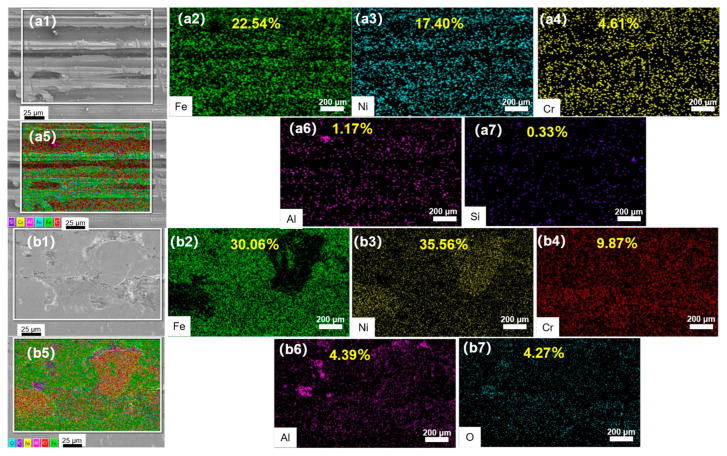
EDS surface scan element distribution of 0.3 MPa specimen with different rolling depths: (**a1**–**a7**) no ultrasonic rolling, (**b1**–**b7**) rolling depth of 0.085 mm.

EDS diagrams (Figure 3) present the distribution of the NiCrAl coating’s elements. Figure 3a,b presents that UR significantly improves the homogeneity of elemental distribution across the coating surface when compared with non-ultrasonic rolling. The internal phase of the material is more uniform, and the internal stress is more average, which directly contributes to strengthening the physical properties of the NiCrAl coating surface.

The accumulation of Cr elements around the crack is more obvious, and the flat surface has a denser distribution of Fe elements (Figure 3(b5)). This phenomenon likely originates from the preferential oxidation of Cr during laser cladding, where Cr readily combines with O elements to produce oxides such as Cr_2_O_3_. The oxides are easy to decompose at high temperatures, allowing oxygen to escape and forming gas inclusions within the interior, which in turn help to form cracks. Ultrasonic rolling, through high-frequency impact on the surface of the coating, causes plastic deformation of the surface material, filling the gap and enhancing the coating’s integrity. Simultaneously, the mass fraction of Ni, Fe, Al, and other elements is higher than that of the non-ultrasonic rolling sample. This apparent increase is likely attributed to the refinement process that the deformation fragments the original dendritic microstructure into a layer of fine and ultrafine grains, which may alter the elemental distribution at the surface and reduce potential porosity.

## 4. Results and Discussion

### 4.1. Effect of Rolling Depth on Microhardness

**Figure 4:**
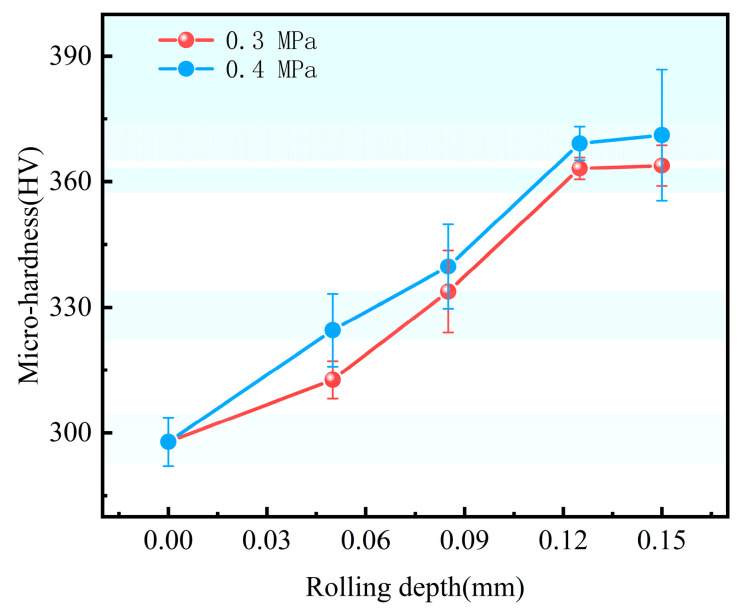
Hardness values at different rolling quantities.

Figure 4 presents the surface hardness evolution of NiCrAl coatings subjected to ultrasonic rolling treatment with varying processing depth. The hardness measurements demonstrate a significant enhancement with the increase in the rolling depth, rising from 297.9 HV (untreated specimen) to 371.1 HV (maximum processed condition), representing a 24.6% improvement in average surface hardness. This strengthening can be attributed to severe plastic deformation induced by high-frequency ultrasonic impacts, which generates dense crystal defects (dislocations, twins, etc.) and subsequent dynamic recrystallization. The continuous formation of subgrain boundaries through this process leads to substantial grain refinement, consistent with the Hall–Petch formula, where decreased grain size directly correlates with increased material hardness.

Furthermore, comparative analysis reveals that specimens processed under 0.4 MPa static load exhibit marginally higher surface hardness than those treated at 0.3 MPa under equivalent rolling depth. This observation confirms that elevated static load contributes to additional grain refinement, thereby further enhancing surface mechanical properties through microstructural modification.

In summary, in the process of ultrasonic rolling, under the same setting conditions, there is an optimal value of the depth of roller head pressing, which can make the hardness of NiCrAl coating reach the highest, and increasing the static load value can also improve the hardness of the coating surface.

### 4.2. Effect of Rolling Depth on Residual Stress

**Figure 5:**
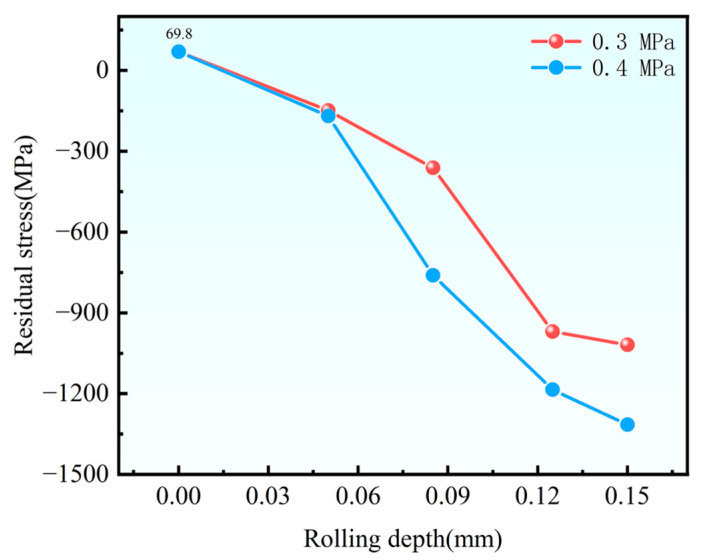
Residual stress values under different rolling amounts.

Figure 5 illustrates the evolution of surface residual stress in NiCrAl coatings as a function of rolling depth. The residual stress exhibits a pronounced increasing trend when the rolling depth rises from 0.05 mm to 0.15 mm. At the minimal rolling depth (0.05 mm), the residual stress remains relatively low (−149.3 MPa), likely due to the insufficient plastic deformation to induce grain refinement. However, a sharp increase in residual stress is observed with greater rolling depth, attributable to intensified microstructural modification under the combined action of static load and high-frequency impact, increasing the residual stress. This phenomenon correlates with enhanced dislocation density and grain boundary formation associated with severe plastic deformation. Notably, the consistent stress trends observed under different static load conditions (0.3 MPa and 0.4 MPa) validate the reproducibility of the experimental results, confirming the reliability of the proposed mechanism.

### 4.3. Effect of Rolling Depth on Surface Roughness

**Figure 6:**
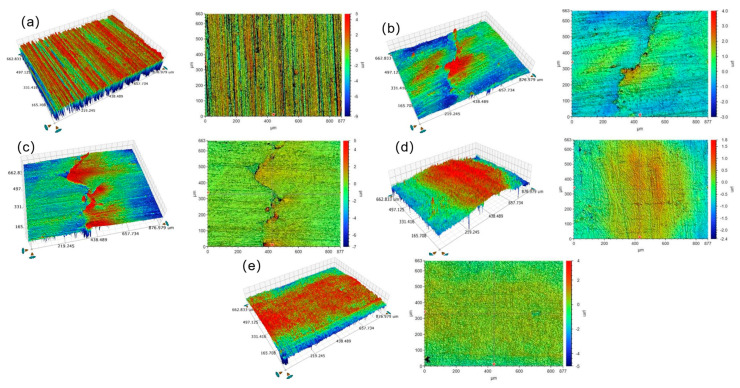
Three-dimensional morphology of laser-clad coating treated with different downward pressures at 0.3 MPa: (**a**) no ultrasonic rolling, (**b**) rolling depth of 0.05 mm, (**c**) rolling depth of 0.085 mm, (**d**) rolling depth of 0.125 mm, (**e**) rolling depth of 0.15 mm.

Figure 6 presents a comparative analysis of the three-dimensional morphology in both X and Y directions for the laser-clad coating, contrasting untreated specimens with those subjected to ultrasonic rolling treatment. The morphological characterization reveals that untreated coatings exhibit substantial roughness with non-uniform distribution, while ultrasonically rolled specimens demonstrate progressively smoother surfaces as rolling depth increases, showing both reduced roughness values and improved uniformity. The surface improvement mechanism can be attributed to the high-frequency vibration of the rolling head, which induces plastic flow of surface material, flattening the micro-peak of the sample surface, and filling the crack, so the specimen surface is polished, and the surface microstructure is improved.

**Figure 7:**
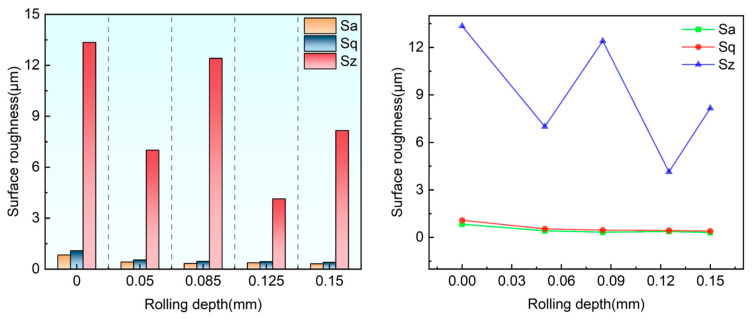
Effect of pressing amount on roughness Sa, Sq, and Sz at 0.3 MPa.

The plastic flow of the metal surface layer facilitates grain refinement and increases the dislocation density. The subsequent slip, accumulation, and rearrangement of the displacement promote the formation of a small-angle grain boundary, thus enhancing the mechanical properties of the specimen surface, including reduced roughness and improved wear resistance. As evidenced in Figure 7, the specimen with a rolling depth of 0.15 mm exhibits superior surface smoothness when other parameters are held constant. This treatment results in the most significant improvement in surface roughness, demonstrating the effectiveness of ultrasonic rolling in surface optimization.

### 4.4. Effect of Rolling on High Temperature Oxidation Resistance

The specimens were subjected to 800 °C isothermal oxidation experiments, with key experimental parameters and oxidation weight gain data summarized in Table 3. A progressive reduction in unit oxidation weight gain was observed with increasing rolling depth, which indicates that the coating’s high-temperature oxidation resistance performance is gradually improving, and based on standard oxidation resistance classification criteria (according to HB5258-2000 criteria [29], materials exhibiting an average oxide spallation mass less than 1.0 mg/cm^2^ are classified as having complete oxidation resistance, whereas samples showing values between 1.0 and 10.0 mg/cm^2^ demonstrate partial oxidation resistance), the untreated coating exhibited antioxidant grade performance, while the ultrasonically rolled coating achieved the complete antioxidant grade status.

**Figure 8:**
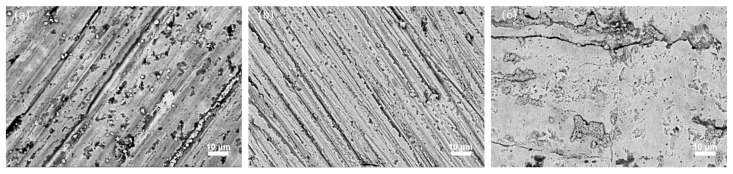
Surface morphologies of three different specimens oxidized at 800 °C for 100 h: (**a**) no ultrasonic rolling, (**b**) rolling depth of 0.05 mm, (**c**) rolling depth of 0.085 mm.

Figure 8 presents the morphology of NiCrAl coatings after high-temperature oxidation. The untreated specimen (Figure 8a) shows numerous scratches and pores, with a thin, discontinuous oxide film that fails to effectively hinder the penetration and diffusion of oxygen elements. In contrast, specimens processed with 0.05 mm rolling depth demonstrate significantly reduced surface defects and a more continuous, dense oxide layer. Further improvement is observed at 0.085 mm rolling depth, where the oxide film appears relatively complete with only a few scratches and oxide shedding.

These observations align with the findings of Niidam et al. [30], who reported that the main products of high-temperature oxidation of NiCrAl coatings are Al_2_O_3_ and Cr_2_O_3_, and the formation of dense and continuous Al_2_O_3_ and Cr_2_O_3_ on the specimen surface provides exceptional surface protection against oxidative degradation.

**Figure 9:**
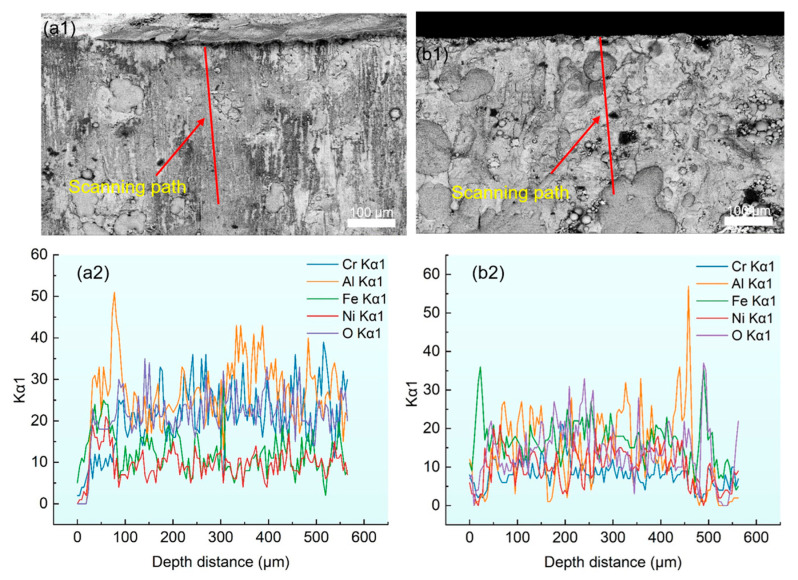
EDS element line scanning of specimens: (**a1**,**a2**) no ultrasonic rolling, (**b1**,**b2**) ultrasonic rolling depth of 0.05 mm.

Figure 9 presents the EDS line scanning results of the NiCrAl coating. The analysis reveals a significant spatial overlap in the enrichment zone of Ni and Fe elements, indicating that Fe-Ni compounds are generated inside. The presence of Ni elements ensures excellent comprehensive properties of nickel-based superalloys by forming *γ*-phase and *γ*′ phase duplex structures with other elements.

The EDS cross-section line scan of the sample with the ultrasonic rolling depth of 0.05 mm showed that the content of Cr was relatively low, and the content of Al accounted for the majority, indicating that the generated oxide is mainly Al_2_O_3_, so that the generated internal oxide content is reduced. This indicates that the oxide film on the surface of the coating blocks the permeation and diffusion of oxygen elements and directly protects the coating.

However, while EDS is effective for elemental analysis, it cannot probe the crystal structure or defect chemistry of the oxide phase. To unambiguously determine the structure and defect populations within this protective Al_2_O_3_ layer, future work should incorporate micro-Raman spectroscopy. As demonstrated in studies of oxide films, Raman spectroscopy [31] is a powerful technique that can not only unambiguously identify the oxide phase (e.g., differentiating between α-Al_2_O_3_ and γ-Al_2_O_3_) but also characterize its point defect populations via defect-induced Raman modes. The type and concentration of these defects are critical factors controlling the diffusivity of oxygen ions through the oxide layer and, consequently, its overall effectiveness as a diffusion barrier.

## 5. Conclusions

This study prepared a NiCrAl coating on a 45# steel substrate using laser cladding technology and compared the performance of the NiCrAl coating after different rolling depths and static load of ultrasonic impact treatments. Based on the above discussions, several key findings can be drawn:

(1) Ultrasonic rolling effectively refines grains and eliminates surface cracks through severe plastic deformation. The elemental distribution becomes more homogeneous, enhancing mechanical properties.

(2) The microhardness of the USRP-treated coating increases by 24.6% (from 297.9 HV to 371.1 HV) compared to the untreated sample, primarily due to grain-boundary strengthening and dislocation accumulation, as governed by the Hall–Petch relationship.

(3) Under constant static load, deeper rolling depths progressively convert surface tensile stress to compressive stress. Higher static load further amplifies compressive stress.

(4) The coating’s high-temperature oxidation resistance performance is gradually improving with deeper rolling depth and larger static load of USRP. USRP transforms the coating’s oxidation resistance from anti-oxidation grade to full anti-oxidation grade at 800 °C. The denser and more continuous Al_2_O_3_-rich oxide layer reduces internal oxidation, significantly inhibiting oxygen diffusion.

## Figures and Tables

**Table 1:** Chemical composition of NiCrAl powder (mass fraction%).

Element	Cr	Al	Ni
percentage	17	5	Bal.

**Table 2:** Sample parameters of laser cladding.

Laser Power (W)	Scanning Speed (mm/s)	Laser Spot Straight (mm)	Shield Gas Flow Rate (L/min)	Overlap Rate (%)
800–1300	5	3.5	15	50

**Table 3:** Unit oxidation weight gain data of three specimens in the 800 °C isothermal oxidation experiment.

Time (h)	25	50	75	100
No ultrasonic rolling	0.628 mg/cm^2^	0.832 mg/cm^2^	0.982 mg/cm^2^	1.055 mg/cm^2^
Rolling depth of 0.05 mm	0.589 mg/cm^2^	0.785 mg/cm^2^	0.889 mg/cm^2^	0.946 mg/cm^2^
Rolling depth of 0.085 mm	0.578 mg/cm^2^	0.762 mg/cm^2^	0.862 mg/cm^2^	0.926 mg/cm^2^

## Data Availability

The original contributions presented in this study are included in the article. Further inquiries can be directed to the corresponding authors.
